# Notable Programs in Neurotology Series: The University of Miami Ear Institute

**DOI:** 10.1097/ONO.0000000000000063

**Published:** 2024-12-09

**Authors:** Allison D. Oliva, Simon I. Angeli

**Affiliations:** 1 Department of Otolaryngology-Head and Neck Surgery, University of Miami Miller School of Medicine, Miami, FL

**Keywords:** History of otology, Neurotology, Otology

## Abstract

The University of Miami Ear Institute (UMEI) was conceived and founded by Dr. W. Jarrard (Jerry) Goodwin in 1990, then Chairman of the University of Miami Department of Otolaryngology–Head and Neck Surgery. Dr. Goodwin’s goal was to establish a state-of-the-art institution featuring world-renowned experts in otology, audiology, cochlear implants, balance disorders, skull base surgery, and research. With the support of many within and outside the University, he succeeded in this endeavor and appointed Thomas J. Balkany the first director of the Ear Institute. Under Dr. Balkany’s leadership, the institute continued to evolve alongside the growing University of Miami Department of Otolaryngology, pioneering developments in pediatric cochlear implant surgery and postoperative care and basic science research. Dr. Balkany transitioned from UMEI Director in 2010, succeeded by Dr. Fred F. Telischi, and subsequently Dr. Simon I. Angeli who is the current Ear Institute Director. The Ear Institute experienced exponential growth in clinical services, research, education, and advocacy throughout the 2nd decade of the 21st century. Renamed the UHealth Ear Institute, its organizational structure evolved to meet new challenges. In its 34-year history, the UHealth Ear Institute has transformed from an idea into a nationally and internationally recognized center of excellence. It remains dedicated to advocating for universal hearing health, ensuring access to hearing health services, providing exceptional patient care, advancing innovative research, and training future specialists.

The University of Miami Ear Institute (UMEI) was conceived and founded by Dr. W. Jarrard (Jerry) Goodwin in 1990, then Chairman of the University of Miami Department of Otolaryngology–Head and Neck Surgery (Fig. 1). Dr. Goodwin’s goal was to establish a state-of-the-art institution featuring world-renowned experts in otology, audiology, cochlear implants, balance disorders, skull base surgery, and research. He lobbied the University of Miami Miller School of Medicine (UMMSM) leadership vigorously for space and funding. One influential figure in these fundraising efforts was Joan Nielsen, a magazine publisher and hearing-impaired patient, who recognized the community’s needs and initiated the Ear Dears fundraising organization. This organization played a crucial role in garnering philanthropic support essential to achieving the institute’s financial goals. Early contributions also came from the South Florida Lions Club and the Pilots Club International. Dr. Goodwin, along with Mr. Cesar Ortiz (the department’s administrator) and partners from the UM Development Office, navigated numerous challenges. The UMEI marked its inauguration with the Ear Dears Ball in 1990. Through these collaborative efforts and with the backing of UMMSM Dean Dr. Bernie Fogel, the UMEI was established, with Dr. Thomas Balkany subsequently appointed as its director.

Thomas Balkany, MD, a Miami native, graduated from the UMMSM in 1972. He completed surgical residency at St. Joseph Hospital in Denver, otolaryngology residency at the University of Colorado School of Medicine, and a neurotology fellowship under William House at the House Ear Institute in Los Angeles. After a successful otology-neurotology and cochlear implant practice in Denver, he returned to the University of Miami in 1990 as the founding director of the Ear Institute and cochlear implant program. Dr. Balkany’s career has been distinguished by significant contributions to patients, students, and colleagues. In 2000, he became the Hotchkiss Professor and Chair Emeritus of the Department of Otolaryngology at the UMMSM. The Hodgkiss endowment was given by Miriam Hodgkiss in honor of her husband Walter, who was thought to be the first otolaryngologist in South Florida. Dr. Balkany has received multiple awards for his advancements in cochlear implantation and ethical treatment of deaf patients. Over 25 years at the University of Miami, Dr. Balkany restored hearing to over 2000 deaf individuals from South Florida and beyond, spanning regions such as Latin America, Europe, Asia, and the Middle East. He is the founding chairman of the William House Cochlear Implant Study Group and a founding board member of the American Cochlear Implant Alliance (ACIA). Dr. Balkany holds 14 U.S. and international patents on cochlear implant technologies and has authored 3 books and over 300 scientific publications on ear surgery. In 2012, he established the Institute for Cochlear Implant Training, a Florida nonprofit corporation offering advanced courses for cochlear implant surgeons, audiologists, and language specialists worldwide.

## THE EARLY BEGINNINGS

UMEI initially occupied the third floor of the Ambulatory Care Center East on the Jackson Memorial-UM campus in downtown Miami. Early recruits included Dr. Fred F. Telischi, who joined after completing his neurotology fellowship at the House Ear Clinic, and Dr. Annelle Hodges, the institute’s first cochlear implant audiologist in 1992. Drs. Brenda Lonsbury-Martin and Glenn Martin were pivotal hires for directing research, bringing along significant grants. This husband-and-wife research team pioneered otoacoustic emissions research, revolutionizing diagnostic audiology and neonatal hearing screening. Another influential husband and wife recruited to the Ear Institute were Drs. Ron Tusa (oto-neurologist) and Susan Herdman (vestibular therapist). They were nationally recognized for their contributions to the understanding of the diagnosis and management of vestibular disorders. The institute also secured space to establish a state-of-the-art temporal bone dissection laboratory, a cornerstone for training courses in otology and cochlear implant surgery for residents and international ENT physicians, taught by institute surgeons and guest professors. Alongside clinical and research efforts, Drs. Balkany and Hodges championed the UMEI’s mission, advocating for hearing and cochlear implant services statewide.

## THE COCHLEAR IMPLANT PROGRAM

While cochlear implants are now commonplace for restoring hearing in children, this was not the case in the early 1990s. Drs. Balkany and Hodges faced challenges from the deaf community, insurance companies, and fellow clinicians. Dr. Balkany aimed to pioneer cochlear implantation for deaf infants, establishing infrastructure and protocols to support them through surgery, device programming, and language development. Demonstrating the health, social, and psychological benefits of this technology to the community was critical. The outcomes from UMEI, alongside other national and international cochlear implant centers, were instrumental in U.S. Food and Drug Administration approval for infant cochlear implant surgery in the 1990s (1). Today, UMEI surgeons have performed over 3000 cochlear implant surgeries, with over 200 conducted annually. To meet growing clinical demands, pre- and postimplantation services expanded significantly. Dr. Hodges, through industry grants, led a clinical fellowship training program for cochlear implant audiologists, addressing diagnostic and programming needs at UMEI and other centers.

As expected, the rapid growth in cochlear implant surgeries revealed the gap in services for nonimplanted children. To address this gap, the Ear Institute established the Children’s Hearing Program (*aka* CHP) in 2014. Kari Morgenstein AuD was appointed Director of CHP, and immediately began the work of assembling a comprehensive pediatric program that would serve the entire age and severity spectrum of pediatric hearing loss. Dr. Morgensein established strong collaborations with community providers, policy makers and birthing hospitals to secure the program continuity and patients’ access to all services. Leadership of CHP transitioned to Chrisanda Sanchez AuD in May 2020, and she has successfully led the program since then, managing up to 7 pediatric audiologists and training of future pediatric audiologists.

A grant from Mr. Barton G. Weiss, a successful Miami restauranteur and the father of a cochlear implant child, established the Barton G. Weiss Kids Hear Now Center. Not knowing how to navigate the hearing loss journey and feeling alone in the process, Mr. Weiss launched the Kids Hear Now Foundation in 2008 along with Mrs. Jill Viner. In 2010, he partnered with the University of Miami Ear Institute and established the Barton G Kids Hear Now Cochlear Implant Family Resource Center. As part of this partnership, Kids Hear Now pledged to donate 5 million dollars to support a multidisciplinary team that would staff the Family Resource Center. The center offers a broad array of services for pediatric cochlear implant patients and families including family counseling and mental health services, parental education to ensure listening and spoken language development, emotional support during surgery, and mentoring programs. The Center also organizes summer camps and round-the-year family events for implanted children and their families. The power of our interdisciplinary teams allows us to cotreat with families and obtain a better understanding of their expectations and needs. Our program also has 2 loaner programs: Oliver Hearing Aid Bank and the Sharing Auditory Miracles Fund for cochlear implants. In addition, the Center takes pride in assisting international families who are uninsured or do not have access to cochlear implants in their home country, and we perform up to 2 pro-bono cochlear implant surgeries per year with the support from industry and community partners. Together, with support from local and national organizations, we ensure that deaf or hard of hearing individuals are not left without sound. Leading these efforts is Ivette Cejas, PhD, a Psychologist and Director of Family Support Services at the Ear Institute. Dr. Cejas has published extensively on parenting support and other socioeconomic and wellness factors influencing outcomes in cochlear implantation (2). She and her CHP colleagues have continued the well-known Childhood Development after Cochlear Implantation longitudinal cohort study of early implanted children and their development.

## RESEARCH AND EDUCATION EXCELLENCE

Initially supported by grants from cochlear implant manufacturers, the Ear Institute conducted multi-institutional clinical trials involving many patients. Thomas van de Water, PhD, led the basic sciences laboratory in 2000, focusing on cochlear implant insertion trauma. Adrien Eshraghi, MD, published groundbreaking work on insertion trauma and otoprotection. Dr Eshraghi’s new grading system of inner ear trauma was widely adopted by research centers around the world (3). Drs. Van de Water and Eshraghi developed the first dexamethasone eluding cochlear implant electrode that is currently undergoing clinical trials (4). Suhrud Rajguru, PhD, joining in 2011, demonstrated stimulation of inner ear cells and dendrites using infrared light, leading to advancements in cochlear implant technology. Collaboration under Dr. Rajguru’s leadership resulted in a hypothermia probe to mitigate cochlear insertion trauma (5). Basic science research on otoprotection informed translational studies, including the development of pharmacological protocols like the “Miami Cocktail” for hearing preservation in cochlear implantation (6). Successful outcomes in preserving post-implantation hearing facilitated combined stimulation with hearing aids and cochlear implants in the same ear. In 2016, Meredith Holcomb AuD joined as Cochlear Implant Program Director, overseeing its evolution with a team of specialized audiologists. Standardization of practices, expansion to satellite clinics, outcomes data collection, multidisciplinary services, and clinical research efforts led to the Cochlear Implant Program’s designation as a Center of Excellence (7).

## PRESENT DAY

Dr. Balkany transitioned from UMEI Director in 2010, succeeded by Dr. Fred Telischi, and subsequently Dr. Simon Angeli who became the current Ear Institute Director when Dr. Telischi became Department Chairman in 2011 (Fig. 2). The Ear Institute experienced exponential growth in clinical services, research, education, and advocacy throughout the 2nd decade of the 21st century. Renamed the UHealth Ear Institute, its organizational structure evolved to meet new challenges. Hillary Snapp, PhD, recognized for her work in bone auditory implants (8), was appointed Director of the Audiology Division in 2018, achieving notable milestones such as UHealth audiologists gaining UM faculty status and advancing as independent researchers. Best practices in diagnostic audiology, hearing aid fitting, and tinnitus management were standardized under her leadership. UHealth clinician-scientists continue to lead innovative research in ear and auditory pathways. Michael Hoffer MD brought expertise and grants from his 20-plus years in the US Navy, focusing on mild Traumatic Brain Injury, tinnitus, and vestibular disorders (9). Christine Dinh, MD, developed a translational research pipeline for testing novel therapies in vestibular schwannoma and neurofibromatosis 2 (NF2), led the multidisciplinary team that established the University of Miami Auditory Brainstem Implant Program (10), and is the site investigator of the INTUITT-NF2 Trial (11). Juan Chiossone, MD, directs the Canes Laboratory, a state-of-the-art microsurgery training center inaugurated in 2018, replacing the original temporal bone dissection laboratory, and advancing the institute’s educational mission (12). Vivek Kanumuri, MD, the latest addition to the neurotology team, investigates neural mechanisms of tinnitus. Under the steadfast leadership of Dr. Fred Telischi, the Department’s Chairman, the Department of Otolaryngology–Head and Neck Surgery and the UHealth Ear Institute have experienced considerable growth in recent years. In the fiscal year 2023–2024, 85,898 patient clinic visits were recorded, 5861 surgeries were performed, over 130 peer-reviewed articles and book chapters were published, and $3.972 million in research grants were secured, placing the Department at No. 23 in the Blueridge NIH funding national rank. Xue Zhong Liu, MD, PhD, Director of the Miami Ontogenetic Program and consistently among the top 10 NIH funded otolaryngologists in the US, contributed significantly to these achievements.

Our surgeons have developed significant multidisciplinary collaboration with clinicians and scientists from the University of Miami and other institutions. Many have dual appointments in Neurosurgery and Pediatrics. Our surgeons cofounded with members of the Neurosurgery, Radiology, Radiation Oncology, Pathology, Endocrinology, as well as surgeons from the divisions of Rhinology and Head and Neck Surgery, the first institutional tumor board focused on skull base tumors called multispecialists of the base of skull. Collaborations with the Nicklaus Children’s Hospital and the Mailman Center for Child Development have been impactful for the development of hearing screening and early intervention services. Many of our surgeons have distinguished volunteer careers as leaders in scientific societies. Many have served as Senior Examiner of the American Board of Otolaryngology and the American Board of Neurotology, been members of the Boards of Directors of the American Academy of Otolaryngology–Head and Neck Surgery, Auditory-Verbal International, AG Bell, the Florida Society of Otolaryngology–Head and Neck Surgery. To name a few, Dr. Telischi was president of the American Neurotology Society, Dr. Hoffer is president of the Triological Society, Dr. Angeli was treasurer of the Panamerican Association of Otorhinolaryngology, Dr. Holcomb was the first woman and first audiologist to chair the ACIA, and Dr. Guerreiro is a Director of the Florida Board of Speech-Language Pathology and Audiology.

An integral part of the Ear Institute is the neurotology fellowship program, with the first fellow graduating in 1996. There were 2 parallel fellowship programs with similar curriculum and training, one approved by the ACGME, and the International Neurotology fellowship. This latter was open to international otolaryngologists who sought to pursue training in neurotology and lateral skull base surgery. Graduates from Colombia, Jamaica, India, New Zealand, Turkey, Nepal, Bahrain, Saudi Arabia, Lebanon, Jordan, the Philippines, Iran, and Israel have returned to their native countries, taken on leadership positions in their national and regional scientific societies, and changed the lives of many patients with their learned skills. In collaboration with the Panamerican Association of Otorhinolaryngology, American Academy of Otolaryngology–Head and Neck Surgery, and several other national and international societies, our observational “mini-fellowship” program has been active since 2001 and contributed to the training of dozens of physicians seeking to improve their otology–neurotology education.

Part of the larger mission of the Ear Institute is to promote diversity, equity, inclusion, and belonging in all areas of function including clinical care, leadership, education, hiring, and interactions with colleagues within the department of otolaryngology and other University of Miami departments. With a varied and balanced gender and cultural representation, our fellows, staff and faculty members continually update our cultural sensitivity, responsiveness, and competency as this is paramount in continuing to provide exceptional care for our patients and where everyone is heard, supported, included, and encouraged.

The mission of the UHealth Ear Institute is to deliver outstanding, personalized, and compassionate care to adults and children with hearing and balance disorders. We achieve this through our steadfast commitment to our core values and a focus on patient-centered care, impactful research, sensibility to diversity, and medical education. Continuing Dr. Balkany’s legacy, the UHealth Ear Institute has grown and evolved into a nationally and internationally recognized center of excellence and remains dedicated to advocating for universal hearing health, ensuring access to hearing health services, providing exceptional patient care, advancing innovative research, and training future specialists.

**Figure 1.**
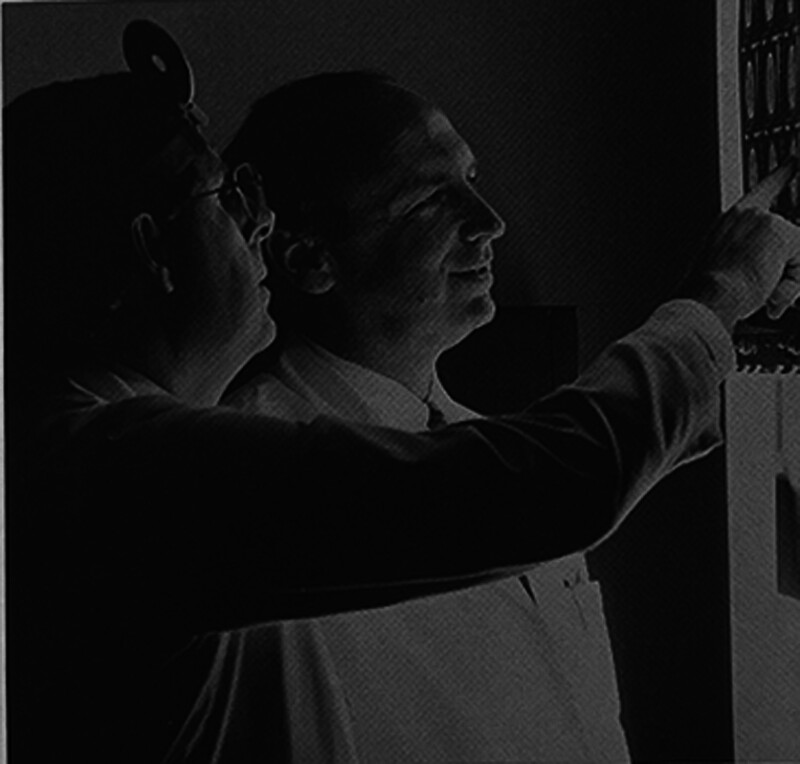
Dr. Thomas Balkany, then Director of the University of Miami Ear Institute, and Dr. Jarrard Goodwin, then chairman of the University of Miami Department of Otolaryngology, review imaging at the Ear Institute (1995). Image courtesy of the University Archives, University of Miami Libraries, Coral Gables, Florida.

**Figure 2.**
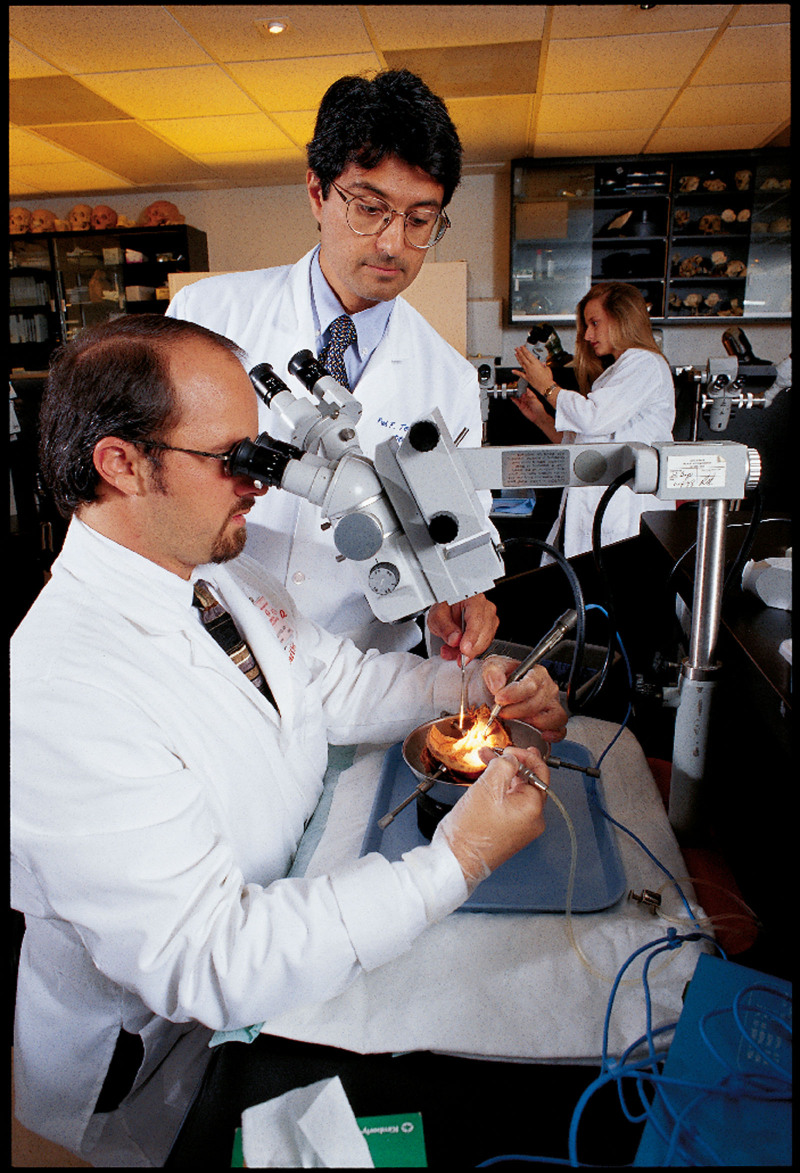
Dr. Fred Telischi instructs then University of Miami Neurotology fellow, Dr. Adrien Eshraghi in the original temporal bone dissection laboratory (2001). This was later replaced by the CANES laboratory in 2018. Image Courtesy of the University Archives, University of Miami Libraries, Coral Gables, Florida.

## ACKNOWLEDGMENTS

The authors are immensely grateful to the following persons for their invaluable contributions to this article: Dr. W. Jarrard Goodwin, Dr. Thomas J. Balkany, Dr. Anelle Hodges, Dr. Fred F. Telischi, and Mr. César Ortiz.

## FUNDING SOURCES

Funding for this project was provided by the University of Miami Department of Otolaryngology–Head and Neck Surgery.

## CONFLICT OF INTEREST STATEMENT

The authors have no conflict of interest to disclose.
